# Is 'Opt-Out HIV Testing' a real option among pregnant women in rural districts in Kenya?

**DOI:** 10.1186/1471-2458-11-151

**Published:** 2011-03-08

**Authors:** Opondo Awiti Ujiji, Birgitta Rubenson, Festus Ilako, Gaetano Marrone, David Wamalwa, Gilbert Wangalwa, Anna Mia Ekström

**Affiliations:** 1 Karolinska Institutet, Department of Public Health Sciences, Division of Global Health, Stockholm, Sweden; 2 African Medical Research Foundation (AMREF) in Kenya, P.O. Box 30125-00100, Nairobi, Kenya; 3 AMREF Busia Child Survival Project (BCSP), P.O. Box 30125-00100, Nairobi, Kenya; 4 Karolinska University Hospital, Department of Infectious Diseases, Stockholm, Sweden

## Abstract

**Background:**

An 'opt-out' policy of routine HIV counseling and testing (HCT) is being implemented across sub-Saharan Africa to expand prevention of mother-to-child transmission (PMTCT). Although the underlying assumption is that pregnant women in rural Africa are able to voluntarily consent to HIV testing, little is known about the reality and whether 'opt-out' HCT leads to higher completion rates of PMTCT. Factors associated with consent to HIV testing under the 'opt-out' approach were investigated through a large cross-sectional study in Kenya.

**Methods:**

Observations during HIV pre-test information sessions were followed by a cross-sectional survey of 900 pregnant women in three public district hospitals carrying out PMTCT in the Busia district. Women on their first antenatal care (ANC) visit during the current pregnancy were interviewed after giving blood for HIV testing but before learning their test results. Descriptive statistics and multivariate regression analysis were performed.

**Results:**

Of the 900 women participating, 97% tested for HIV. Lack of testing kits was the only reason for women not being tested, i.e. nobody declined HIV testing. Despite the fact that 96% had more than four earlier pregnancies and 37% had been tested for HIV at ANC previously, only 17% of the women surveyed knew that testing was optional. Only 20% of those surveyed felt they could make an informed decision to decline HIV testing. Making an informed decision to decline HIV testing was associated with knowing that testing was optional (OR = 5.44, 95%CI 3.44-8.59), not having a stable relationship with the child's father (OR = 1.76, 95%CI 1.02-3.03), and not having discussed HIV testing with a partner before the ANC visit (OR = 2.64 95%CI 1.79-3.86).

**Conclusion:**

High coverage of HIV testing appears to be achieved at the cost of pregnant women not understanding that testing is optional. Good quality HIV pre-test information is central to ensure that pregnant women understand and accept the reasons for testing and will thus come back to collect their test results, an important prerequisite for completing PMTCT for those who test HIV-positive.

## Background

The World Health Organization (WHO) and the joint United Nations program on HIV/AIDS (UNAIDS) revised the guidelines for HIV testing in 2007 [1]. The current guidelines were designed to increase coverage of testing and identify patients in need of antiretroviral therapy (ART). In the former 'opt in' HIV strategy, the initiative to be tested was with the individual, not with the health care services, and individual pre-test counseling followed by informed consent was required before testing. In some areas, people were even required to sign a separate informed consent form, which detailed the risks and benefits of being tested [2]. With the new 'opt-out' strategy, individuals have to actively opt out or decline the HIV test after a pre-test information session, often carried out in a group, while post-test counseling is still carried out on an individual basis for all clients.

The implications of provider-initiated HIV testing greatly affect women in sub-Saharan Africa (SSA) where they account for nearly 60% of those infected with HIV and where 75% of those living with HIV are between 15-24 years [3]. Women have more contact with the health services e.g. during pregnancy [4] and are thus more likely to undergo HIV screening [5], but it has been observed that consent may be compromised in SSA, which negatively affects women's autonomy and possibly also completion of PMTCT [6,7].

The shift from 'opt in'/client-initiated to 'opt out'/provider-initiated HIV testing has generated a debate on how to best increase the uptake of HIV testing while, at the same time, protect individual rights to voluntary consent for HIV testing [1]. Proponents of "opt out" assert that the provider-initiated consent process is crucial to achieve high coverage of HIV testing and prevention of mother-to-child transmissions (PMTCT) while it still protects autonomy [8]. It also helps the 'streamlining' of HIV into 'normal care' thereby decreasing the stigma [8,9].

Those who question the 'streamlined' consent process express doubt about whether informed consent can be ensured in the context of routinely offered HIV testing under conditions of scarce human resources [10,11]. Power differences in the provider-client relationship is also identified as a problem, since it is uncertain whether clients who normally have a lower social status will feel able to opt out of testing against the recommendation of their providers [6]. Others are concerned about the client's ability to provide voluntary consent and to what extent any choice will be presented given that providers are encouraged to motivate clients to test and could be coercive [6]. Women in particular are often also unable to make decisions independently due to gender inequality and lack of knowledge [3,12]. Finally, and most important from a public health perspective, there is concern that pregnant women who fail to make an informed choice about HIV testing are less likely to come back for their test results, an obvious prerequisite for identifying and enrolling HIV-infected women in the PMTCT program, thus undermining the quality and effectiveness of this important intervention [5,13]. A study from Botswana showed that pregnant women felt compelled to test when it was routinely offered and some instead exerted their decision-making power by not returning to collect their test results [13].

Kenya introduced routine rapid 'opt out' HIV testing at antenatal care (ANC) in 2007 [14]. Approximately 76 000 pregnant women are living with HIV in Kenya, thus ranking it sixth among the ten African countries that contribute 67% of the global burden of MTCT [15]. Up to 40% of all pregnant women enrolled in ANC programs in Busia district in western Kenya are estimated to not come back for their test results and will thus never be enrolled into PMTCT (personal communication). Pregnant women and their infants in these two rural districts are considered to be highly vulnerable to MTCT due to the high HIV prevalence (9%) and high fertility rate (7.1) compared to the national average of 7% and 5.1 respectively [14]. This study aims to identify factors associated with consent to HIV testing under the 'opt out' strategy in this area in rural Kenya.

## Methods

### Study area

This study was performed in Busia district located in western Kenya. This rural district has five administrative divisions with a population estimated at 415 000. The study catchment area has a population of 202 348 living in 312 villages, with 50 000 women of reproductive age and 38 000 children less than five years of age. Surveillance studies at ANC show HIV infection rates close to 10% [14]. Agriculture, fishing and small-scale commercial undertakings are the main economic activities in the district where the average household generates approximately $84 per month. The majority ethnic group is Luhya with a few Luo speakers. There are 22 health facilities in the study area that are private, mission-run or government-owned. About 90% of these facilities offer free rapid HIV testing services except for a few dispensaries that refer patients to health centers or district hospitals for testing.

The study was carried out at three public district hospitals collaborating with non-governmental organizations (NGOs) on PMTCT and ART. In all three hospitals, PMTCT and ART have been provided free-of-charge since 2005 to all women in need in line with the WHO treatment guidelines from 2007. According to the new 'opt out' guidelines implemented in 2007, all pregnant women should participate in a HIV pre-test information group session, followed by rapid 'opt out' HIV testing and individual post-test counseling at their first ANC visit. For pregnant women who test positive for HIV, a CD4 cell count is done to determine whether ART should be initiated or if a single dose of nevirapine during labor is enough (short course combination treatment during pregnancy and breastfeeding has not yet been implemented in Busia district as of end 2010). HIV-infected women should be individually counseled regarding hospital delivery, safe infant feeding and contraceptive use.

### Study design, sampling and participants

The study included twelve sit-in observations of counseling sessions for pregnant women and a large cross-sectional survey among pregnant women. The observations were performed by the first author, who is of Kenyan origin and fluent in the local languages spoken in the area, during two randomly selected weekdays and with four visits at each facility.

For the cross-sectional assessment, 900 women who were on their first visit to ANC for the current pregnancy were recruited consecutively between August and December 2008. All women in the three hospitals received the same information during the routine pre-test information sessions that followed the Kenyan guidelines on PMTCT. A midwife informed them about the study in the ANC reception area during a session on general hygiene. Those willing to participate met the midwife, gave informed consent and were enrolled into the study. No woman among those approached declined to participate and no participant had been informed of her HIV test results before the interview. The sample included all pregnant women who were tested in the three hospitals within the timeframe.

### Data collection

Notes were taken during the observations about the setting for the pre-test counseling session, the content of the session and of how the information about HIV testing was given. The interviewer-administered structured questionnaire contained both closed and open-ended questions in Kiswahili or Luhyia. Data was collected on socio-demographic characteristics, relationship factors, awareness and knowledge about MTCT and PMTCT and experiences of the group counseling session and the HIV testing. The Kenya Medical Research Institute (KEMRI) and the regional ethics board of Karolinska Institute approved this study.

### Data analysis

The observations were compared and evaluated against the Kenya pre-test guidelines of the 'opt out' approach after each observed session.

For the cross-sectional data analysis, data were analyzed using SPSS-PASW, version 18 (SPSS, Inc., Chicago, IL). Descriptive statistics were used to summarize all variables of interest in the study population.

The outcome variable "making an informed decision to decline HIV testing" was derived from the question 'If you could choose to HIV test or not, would you decline? (Yes/No)'. Independent variables that were used to model the outcome variable included; *type of union *- 'What is your marital status? (Married/Unmarried)', *duration of current sexual relationship *- 'How long have you been in the current relationship? (Not in a relationship, ≤4 years and >4)', *stable relationship with the child's father *- 'Do you live together with the child's father? (Yes/No)', *knowing HIV testing is optional *- 'Do you know that you can choose to HIV test or not? (Yes/No)', *tested for HIV *- 'Have you tested for HIV at this visit? (Yes/No)', *discussing HIV testing with the partner before the ANC visit *- 'Have you discussed HIV testing with your partner before this ANC visit? (Yes/No) and *knowing testing is performed at ANC before the visit *- 'Did you know that HIV testing is done at ANC before coming today? (Yes/No)'

The estimated prevalence of "making an informed decision to decline HIV testing" was reported as a percentage. It is important to note that the outcome variable "making an informed decision to decline HIV testing" implies making a decision about whether to consent to HIV testing or not after awareness i.e. the actual decision of women who knew that HIV testing is optional as well as the perceived decision among those who did not know that testing is optional, had they known that this was the case.

The association between "making an informed decision to decline HIV testing" and each categorical independent variable was first assessed using Chi-square or, when the number in the contingency tables was too small, Fisher's exact test. Independent variables with a p-value of <0.20 associated with the outcome at the bivariate level were entered into multiple logistic regression models with the exception of age, education and occupation level that were included regardless of p-value in order to adjust for potential residual confounding linked to the main independent variables. Both backwards and forward logistic regression (Wald test) was performed and gave almost similar result. P-values <0.05 (2-sided test) were considered significant in the final model. Odds ratios (ORs) and their 95% confidence intervals (CI) were computed. The final multivariate model was tested for goodness of fit with the Hosmer-Lemeshow test.

## Results

### Observations during pre-test information session at group level

#### The setting of the pre-test counseling session

Pre-test counseling sessions were provided to groups of 10-15 pregnant women in a separate space. There were between three and four information sessions per day at each facility. The sessions normally took 45-50 minutes and were mainly performed in the national language Kiswahili and translated simultaneously into the local dialect of Luhyia. Female midwives greeted the audience and introduced themselves when starting the session. The pregnant women were told that they could ask questions during the session in case they wanted to know more, but no woman asked any question or sought clarification at any of the sessions observed. The pregnant women nodded unanimously when the midwife sought to stress the benefits of HIV testing as shown below.

Midwife: Do you mothers agree that it is important to test for HIV and protect the unborn child?

Women: (nodding) yes (in a group).

#### The content of the pre-test counseling session

The information included a description of HIV and AIDS, modes of HIV transmission from a pregnant woman to their child during and after pregnancy, the importance of HIV testing for a diagnosis, secondary prevention of HIV transmission to uninfected male partners and the PMTCT program (single dose nevirapine tablets for the mother and syrup for the infant during a six week period after delivery; skilled hospital delivery; and options of exclusive breastfeeding or formula feeding).

#### Information provided about HIV testing

The women were given information about the importance of HIV testing and of learning about their HIV status, and also the status of their partner. Women were not required, but encouraged, to bring their partners in to be HIV tested as well. The importance of having an uninfected baby was emphasized as well as the fact that testing was important in the first trimester of pregnancy. In all the sessions the midwives' undertone was motivational and the message was that testing and knowing one's HIV status was the best decision a mother could make for her unborn child. No information was provided stating that it was an individual and voluntary choice of the woman to decline or accept HIV testing. The midwives referred to the women as 'mothers' and emphasized that it was their responsibility to take the HIV test to protect the baby and have a healthy and virus-free child. When asked by the main author about reasons for not requiring women to bring their partners for HIV testing, the midwives said that men who really loved their women normally accompanied them to ANC to test of their own free will and did not need to be asked to come.

### Cross-sectional survey of 900 pregnant women

Table 1 shows socio-demographic characteristics and HIV testing information of the 900 women enrolled in the study.

**Table 1:** Socio-demographic characteristics and HIV related data of 900 pregnant women

Characteristic		'Making an informed decision to decline HIV testing'		Total N (column %)
		N (row %)	N (row %)	
		**Yes**	**No**	
**All women**		**180 (20%)**	**720 (80%)**	**900 (100%)**

Age (years)	≤20	100 (20%)	388 (80%)	488 (54%)
	>20	80 (19%)	332 (81%)	412 (46%)
Number of pregnancies including current	≤4 pregnancies	4 (10%)	36 (90%)	40 (4%)
	>4 pregnancies	176 (20%)	684 (80%)	860 (96%)
In stable relationship with child's father	Yes	151 (19%)	660 (81%)	811 (90%)
	No	29 (33%)	60 (67%)	89 (10%)
Type of union	Married	133 (18%)	589 (82%)	722 (80%)
	Unmarried	47 (26%)	131 (74%)	178 (20%)
Duration of current relationship (years)	Not in a relationship	12 (36%)	21 (64%)	33 (4%)
	≤4	134 (20%)	525 (80%)	659 (73%)
	>4	34 (16%)	174 (84%)	208 (23%)
Formal education (years)	Never in school	29 (22%)	102 (78%)	131 (15%)
	≤8	142 (20%)	572 (80%)	714 (79%)
	>8	9 (16%)	46 (84%)	55 (6%)
Occupation	Employed	31 (20%)	127 (80%)	158 (18%)
	Unemployed	149 (20%)	593 (80%)	742 (82%)
HIV tested	Yes	172 (20%)	700 (80%)	872 (97%)
	No*	8 (29%)	20 (71%)	28 (3%)
Aware of MTCT after pre-test counseling	Yes	161 (19%)	685 (81%)	846 (94%)
	No	19 (35%)	35 (65%)	54 (6%)
Aware of PMTCT after pre-test counseling	Yes	160 (20%)	658 (80%)	818 (91%)
	No	20 (24%)	62 (76%)	82 (9%)
Aware that HIV testing is done at ANC before visit	Yes	106 (16%)	548 (84%)	654 (73%)
	No	74 (30%)	172 (70%)	246 (27%)
Discussed HIV testing with the partner before ANC visit	Yes	101(16%)	520 (84%)	621 (69%)
	No	49 (18%)	230 (82%)	279 (31%)
Knew that HIV testing is voluntary	Yes	71 (47%)	79 (53%)	150 (17%)
	No	109 (15%)	641 (85%)	750 (83%)
Tested for HIV at ANC previously	Yes	68 (20%)	264 (80%)	332 (37%)
	No	112 (20%)	456 (80%)	568 (63%)
Understood pre-test counseling	Yes	140 (17%)	670 (83%)	810 (90%)
	No	40 (44%)	50 (56%)	90 (10%)

* HIV testing kits were lacking

The median age was 20 years (inter-quartile range 5). The majority of the women (96%) had already had more than four pregnancies including the current one, although 73% were in a relationship of less than four years. About 90% had a stable relationship with the child's father. Eighty percent were in a formal union. About 85% had eight or less years of formal education and 18% were employed. Slightly over one-third (37%), had previously been tested for HIV at ANC using the 'opt-out' approach.

Lack of testing kits was the only reason for women not to be tested i.e., no woman declined HIV testing and nearly all were tested for HIV (97%). About 73% knew that HIV testing was done at ANC before coming there and 69% had discussed the test with their partner before the visit. Following the pre-test counseling session, 90% (N = 810) claimed they had understood the information, but only 17% had grasped that HIV testing was optional, 95% were aware of MTCT and 91% had understood that preventing transmission was possible. The reasons given by the 10% (N = 90) women who reported not understanding most of the pre-test information included: the counselor speaking too fast (N = 45), using complicated terms (N = 27) and difficult language (N = 18).

Only 20% (N = 180) of the women said they would make an informed decision to decline HIV testing. After adjusting for all potential confounding factors listed in Table 1, only three factors remained independently associated with an increased likelihood of making an informed decision to decline HIV testing in the final multivariate model: knowing that testing was optional, not having a stable relationship with the child's father and not having discussed HIV testing with a partner before the ANC visit. Knowing that testing was optional was the strongest predictor for women saying that they would make an informed decision to decline HIV testing (OR = 5.44, 95% CI 3.44-8.59). Women not in a stable relationship with the child's father were more likely to perceive that they would make an informed decision to decline HIV testing (OR = 1.76, 95% CI 1.02-3.03). Not having discussed HIV testing with a partner before the ANC visit also doubled the likelihood for women saying that they would make an informed decision to decline HIV testing (OR = 2.63, 95% CI 1.79-3.86).

Age, occupation and education level were not statistically significant factors but kept in the final model to adjust for residual confounding often associated with these fundamental variables (Table 2). The number of pregnancies both as a categorical and a continuous variable was not significantly associated with the outcome, probably because only one third had been HIV tested before (OR = 1.96, 95% CI 0.63-6.09).

**Table 2:** Factors included in the final multivariate model analyzing the association with 'making an informed decision to decline HIV testing' among 900 pregnant women

		Crude analysis		Adjusted analysis
**Factor**		**p-value**	**OR (95% CI)**	**OR (95% CI)**

In stable relationship with child's father	Yes		1	
	No	0.002*	2.11 (1.31 - 3.41)	1.76 (1.02 - 3.03)
Discussed testing with partner before ANC visit	Yes		1	
	No	<0.001*	2.65 (1.89 - 3.71)	2.64 (1.79 - 3.86)
Knew HIV testing is voluntary	No		1	
	Yes	<0.001*	5.29 (3.62 - 7.72)	5.44 (3.44 - 8.59)
Age (years)	≤ 20		1	
	> 20	0.513	1.07 (0.87 - 1.32)	1.09 (0.41 - 2.92)
Occupation	Unemployed		1	
	Employed	0.895	1.03 (0.67 - 1.59)	1.05 (0.65 - 1.71)
Formal education	Never in school		1	
	≤ 8 years	0.542	1.26 (0.60 - 2.63)	1.19 (0.54 - 2.63)
	> 8 years	0.309	1.53 (0.67 - 3.49)	1.49 (0.61 - 3.63)

*p-value < 0.05

## Discussion

None of the 900 pregnant women included in this study declined HIV testing under the routine 'opt-out' approach. A majority (83%) had not understood that HIV testing was optional and only one in five stated that they would have been able to make an informed decision to decline HIV testing. This is a fundamental shortcoming of unclear pre-test information, which undermines the assumption of voluntary consent. Thus with the current approach, high coverage of HIV testing at ANC may be achieved at the cost of women not understanding that testing is optional and at the risk of low uptake and completion of PMTCT which is a major problem not only in this area, where between 30%-40% of all pregnant women enrolled in ANC programs are estimated to not come back for their test results (personal communication by David Wamalwa project manager for Busia Child Survival Project), but also documented in other parts of SSA [16,17].

The midwives did provide correct information regarding the importance of HIV testing in the first trimester of pregnancy, but the great majority of women (83%) never understood that it was optional. By saying that testing was 'the best decision a mother could make for her unborn child' the midwives clearly revealed their expectations and left little room for the women to act otherwise. This finding is consistent with another study performed in Kenya showing that women accept HIV testing so as to avoid being perceived as not accepting the message of the midwives [7]. Our findings showed that it was difficult for the providers to remain neutral when informing about routine HIV testing. During the observed counseling sessions the midwives referred to the women as 'mothers' thereby highlighting the importance of the baby. The reason given during the sessions for having the test was the need to protect the child, while nothing was said about HIV testing being optional. From a public health perspective it is important that the women understand and accept the reasons for testing, since this increases enrolment in and adherence to PMTCT.

The high number of women counseled simultaneously under the opt-out approach made meaningful interaction difficult and for the midwives it was consequently easiest to provide information using a top-down approach. This approach could be justified as it reduced waiting time for the many women visiting ANC, in a situation where pre-test counseling is the first step before receiving other services of ANC. However, the current set-up makes most women believe that HIV testing is a prerequisite for obtaining other ANC services. To avoid this, the information needs to not only discuss the benefits of the testing, but also its implications and the importance of the post-test counseling. Our findings showed that 83% of the women perceived testing as a mandatory part of ANC services, not as a service independent of antenatal care. This finding could be attributed to unclear delivery of pre-test information and is consistent with observations that poor counseling prevents pregnant women from making informed decisions about HIV testing.

Only 20% of the women felt they would have been able to make an informed decision to decline HIV testing. This is a remarkably low proportion, given that more than a third also had been tested for HIV at ANC before. Although none among the 900 women declined the test, a majority seemed to have accepted it because they felt obliged to. An explanation for the misconception could be the power difference between the midwives and the pregnant women. Midwives are trusted and have high social status among pregnant women. In a recent qualitative study exploring reasons for adherence to PMTCT in the same setting we found that HIV-infected pregnant women trusted the midwives to keep their HIV diagnosis secret from the mother-in-law at least during pregnancy appointments (data not yet published).

Our findings showed that a great majority of the women had started childbearing at an early age and that 85% of the women had eight years or less of schooling. Possibly many women accepted to have the HIV test because they perceived the midwife to be more knowledgeable and to know best. They seemed not to understand the importance of their own active involvement in accepting or declining the HIV testing and the consequences of having the test. This is consistent with observations that patients in SSA accept to follow recommendations from health providers without fully understanding the consequences of their action as was observed in, for example, Botswana [13]. The implications for HIV testing could be that pregnant women accept to be HIV tested but fail to return for the test results as they realize that they are unprepared for the consequences. Failure to return for results or drop out from PMTCT has been documented from SSA [16,17]. Unfortunately, we were not able to follow the women through the PMTCT process to assess the completion rate, but as mentioned above, a high proportion of pregnant women in this area are reported to never pick up their test results. The rapid test results are available within a quarter of an hour, and so failure to come for them strongly indicates that many women were not ready to face the consequences of a positive test result. They exerted their decision-making power in a more socially acceptable way by dropping out directly after testing. For improved access to and completion of PMTCT pregnant women need to understand the process of testing and voluntarily and consciously consent to HIV testing [18].

In a qualitative study in the Kibera slum in Nairobi exploring the reasons for becoming pregnant among women on ART, we found that women planned to become pregnant to strengthen their sexual relationships and possibly formalize them [19]. In this study the women who did not discuss with their partners felt more able to decline testing, as did those in unstable relationships. These women live in a more insecure situation and lack support to handle the test results, while those in stable relationships and those who have discussed the testing know that they will have support irrespective of the test-results. Couple testing is often promoted as a means to increase male partner support. However, the state of the relationship between a woman and her partner influences the decision-making of the woman in relation to testing. Perceived negative consequences of an HIV diagnosis, such as partner abandonment, isolation and loss of financial support, may be an important reason for women to test alone, to decline picking-up their test results or to avoid HIV testing altogether. This study shows the importance of having a secure relationship and a supportive environment before the testing. A recent study in rural Uganda showed that pregnant women often feel heavily burdened by partner disclosure and couple testing recommendations in relationships where they feel disempowered and dependant on their male partner [20]. It becomes necessary to understand individual women's sexual relationships and dependencies on men in order to improve acceptance of HIV testing and also enrolment and completion of PMTCT.

The likelihood of selection bias was low since ANC attendance is high in Kenya, about 90% visit ANC at least once, and one can assume that our participants represent of a majority of pregnant women in this area. The hospitals included in this study are NGO-affiliated and one can assume that the quality of care is similar across the PMTCT programs.

## Conclusion

High coverage of HIV testing appears to be achieved at the cost of pregnant women's lack of knowledge that testing is optional. Good quality HIV pre-test counseling is central for making pregnant women understand and accept the reasons for testing and encourage consent to HIV testing, an important prerequisite for the consequent completion of the PMTCT program by those who are HIV infected. While provider-initiated HIV testing is necessary to increase the number of women who access PMTCT and ART, caution must be taken to actively involve the woman during the consent process, to respect their autonomy and improve the enrollment and completion of PMTCT. Intensive community campaigns are warranted to raise awareness of the HIV testing being performed at ANC and the reasons why it is being carried out, to sensitize the community and make them better prepared to make informed decisions. Health authorities could collaborate with NGOs to disseminate information, improve education and increase communication at household level in rural areas to supplement human and material resources shortages. More work is needed to understand how best to develop testing policies that both protect the voluntary consent process and expand testing to increase the implementation of functioning PMTCT-programs in areas with high HIV prevalence in SSA.

## Competing interests

The authors declare that they have no competing interests.

## Authors' contributions

OAU is the main author of the manuscript and involved in all aspects of the study. AME and BR provided scientific expertise and feedback throughout the development of the study and manuscript. FI was involved in the conceptualization of the idea, tool development and reading and editing of the manuscripts. GM provided statistical support, supervised the analysis and edited the paper. GW and DW were involved during preparations and pre-test of the survey, data collection and reading and editing of the manuscript. All co-authors have seen and approved the final version of the paper and have agreed to its submission for publication.

## Pre-publication history

The pre-publication history for this paper can be accessed here:

http://www.biomedcentral.com/1471-2458/11/151/prepub
